# Physics-inspired machine learning of localized intensive properties†

**DOI:** 10.1039/d3sc00841j

**Published:** 2023-04-10

**Authors:** Ke Chen, Christian Kunkel, Bingqing Cheng, Karsten Reuter, Johannes T. Margraf

**Affiliations:** a Fritz-Haber-Institut der Max-Planck-Gesellschaft Faradayweg 4-6 D-14195 Berlin Germany margraf@fhi-berlin.mpg.de; b Chair for Theoretical Chemistry and Catalysis Research Center, Technische Universität München Lichtenbergstraße 4 D-85747 Garching Germany; c Institute of Science and Technology Am Campus 1 3400 Klosterneuburg Austria

## Abstract

Machine learning (ML) has been widely applied to chemical property prediction, most prominently for the energies and forces in molecules and materials. The strong interest in predicting energies in particular has led to a ‘local energy’-based paradigm for modern atomistic ML models, which ensures size-extensivity and a linear scaling of computational cost with system size. However, many electronic properties (such as excitation energies or ionization energies) do not necessarily scale linearly with system size and may even be spatially localized. Using size-extensive models in these cases can lead to large errors. In this work, we explore different strategies for learning intensive and localized properties, using HOMO energies in organic molecules as a representative test case. In particular, we analyze the pooling functions that atomistic neural networks use to predict molecular properties, and suggest an orbital weighted average (OWA) approach that enables the accurate prediction of orbital energies and locations.

## Abstract 2

A physics-inspired machine learning approach to predicting localized intensive properties in molecules is presented. The new method is applied to predicting orbital energies and localisations in potential organic semiconductors.

## Graphical abstract

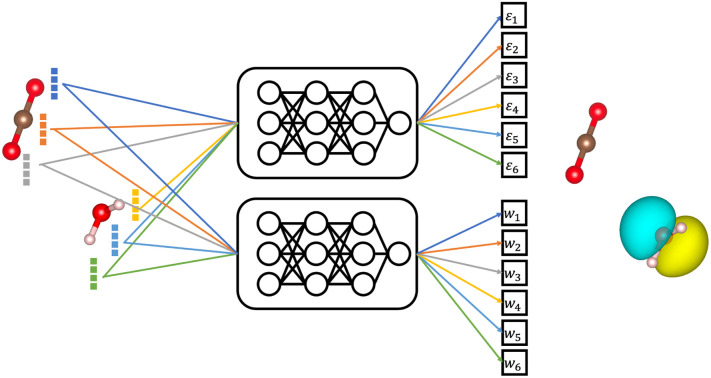

## 1. Introduction

Due to their great potential for accelerating materials discovery and design, there has been significant interest in machine learning (ML) models that enable the fast and accurate prediction of molecular and materials properties.^1–5^ Consequently, a wide range of neural network (NN) and Kernel ML methods have been developed and applied to systems ranging from isolated molecules to complex amorphous solids.^6–14^

**Fig. 1:**
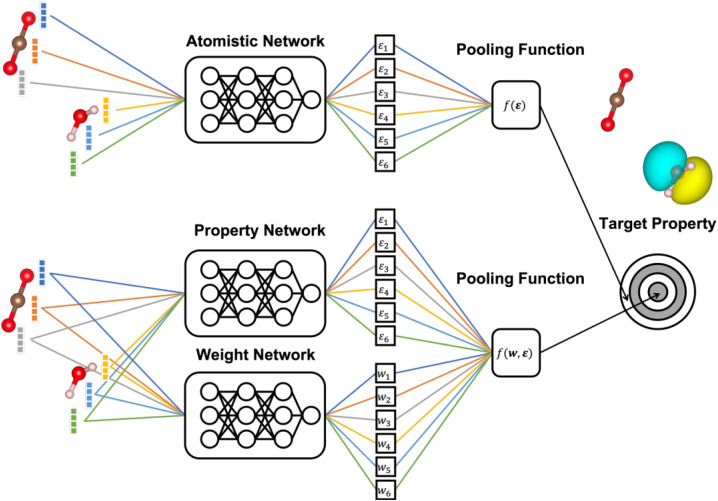
Schematic illustration of atomistic neural networks. In a conventional atomistic neural network (top), the representation of each atomic environment is converted to a scalar output *ε*_*i*_. These outputs are aggregated to the target property using a pooling function. The (orbital) weighted average models introduced herein ((O)WA) additionally predict the weight of each atom in the pooling function, using a second neural network (bottom). This is beneficial in the depicted example case of water and CO_2_, where the target property (in this case an orbital energy) is localized on a part of the system.

In this context, many state-of-the-art approaches exploit the approximately local nature of chemical interactions. This is achieved by representing chemical structures in terms of the element of each atom and the types and positions of the atoms in its immediate surrounding (the chemical environment).^15–17^ This is, *e.g.*, commonly used when developing ML interatomic potentials, where the total energy is then obtained as a sum of local atomic contributions (see Fig. 1).

There are two distinct but related advantages to this approach. On one hand, locality ensures that the computational cost of the model asymptotically displays linear scaling with the size of the system, allowing for instance the routine application of ML potentials to systems with a thousand atoms or more. On the other hand, the summation of atomic contributions ensures size-extensivity, which is often desirable, if not a key requirement as in the case of interatomic potentials.

Simply put, size-extensivity means that predicted properties (*e.g.* energies) scale linearly upon trivial extensions of the system size, *e.g.* when describing ideal crystals in larger periodic supercells or replicating non-interacting molecules. This allows size-extensive ML models to be trained on small molecules or simulation cells and later applied to large systems.^1,16,18^ However, size extensivity is not necessarily always a good assumption. Indeed, many electronic properties like excitation energies,^19^ orbital energies^20^ or ionization potentials^21^ are intensive, meaning that they remain constant for such trivial scalings of the system size. In this case summing over atomic contributions therefore yields unphysical results, in particular when extrapolating to systems that are larger than the ones contained in the training set.

From an ML perspective, the summation of atomic contributions is simply one of many possible pooling functions.^22–24^ For example, when taking the average instead of the sum, predictions remain constant as the system size is scaled.^18,25^ Average pooling is therefore often used as the default pooling function for intensive properties. Unfortunately, average pooling can still yield unphysical results, particularly when the target property is localized and the system has low symmetry.

To illustrate this, consider a model trained on the ionization energies (IEs) of isolated monomers of water (12.6 eV) and CO_2_ (13.8 eV). An average pooling model will correctly predict that the IE remains constant for a non-interacting supersystem consisting of two separated water molecules. However, for a non-interacting supersystem consisting of one water and one CO_2_ molecule, this model would predict that the IE is the average of the corresponding water and CO_2_ values, which is clearly incorrect. The problem here is that the model fails to take into account that an ionization of this supersystem is localized on the water molecule, since it has the lower IE.

While this is a somewhat artificial example, many real chemical systems also display ionizations, excitations or orbitals that are spatially localized. Examples include disordered, defected or doped solids,^26,27^ functionalized organic molecules and polymers,^28^ as well as complex biomolecules like DNA and RNA.^29^ This raises the question whether there are more appropriate pooling functions for electronic properties with a (potentially) localized nature.

In this contribution, we address this question by proposing a series of pooling functions that are formally able to treat localized (electronic) properties correctly. We then develop a new dataset of organic molecules, which is purposefully designed to contain both systems with localized and delocalized highest occupied molecular orbitals (HOMOs). This allows us to extensively benchmark the proposed pooling functions, and analyze their ability to predict the location of the orbital, as well as the energy. Finally, the most reliable methodology is applied to predict the orbital energies of the general OE62 dataset,^30^ consisting of experimentally reported organic molecules with large structural diversity.

## 2. Methods

### 2.1 Atomistic neural networks

The general structure of an atomistic NN is shown in Fig. 1. Briefly, the chemical environment of an atom *i* in a given system with *N* atoms is represented by a vector or tensor *χ*_*i*_. This representation is passed through the NN to yield a scalar output *ε*_*i*_. In a final step, the outputs of all atoms are combined to the global target property *P* through a pooling function *f*(*ε*_1_,…,*ε*_*N*_), to be specified below.

Two classes of atomistic NNs are in common use. The original approach of Behler and Parinello uses a predefined set of radial and angular basis functions to generate the representation of the chemical neighborhood within a fixed cutoff radius around each atom.^15^ Common choices for these predefined representations are the Atomic Symmetry Functions (ASFs) of Behler and Parinello, and the Superposition of Atomic Positions (SOAP) of Bartók and Csányi.^31,32^ More recently, Message-Passing Neural Networks (MPNNs) have been proposed as an alternative.^33,34^ These replace predefined representations with an end-to-end deep NN architecture that learns a data-driven representation during training.

The current paper is focused on the nature of the pooling function and not on the structural representation. For generality, we will therefore consider both approaches in the following. Specifically, the SOAP representation will be used as implemented in Dscribe,^35^ using the universal length scale hyperparameters defined in ref. 36. As a prototypical MPNN, the SchNet architecture is used.^16^ For consistency, both SOAP and SchNet models are implemented with the PyTorch based SchNetPack library,^37^ using default hyperparameters unless noted otherwise (see ESI† for details).

### 2.2 Pooling functions

In the following we focus on learning HOMO energies (*E*_HOMO_) as a prototypical localized intensive property. While the concepts we introduce below are generally applicable to all intensive properties, the concrete shape of the pooling function can vary depending on the target property. Any property-specific aspects will be highlighted when necessary.

The two most commonly used pooling functions in atomistic NNs are sum and average pooling, defined as1
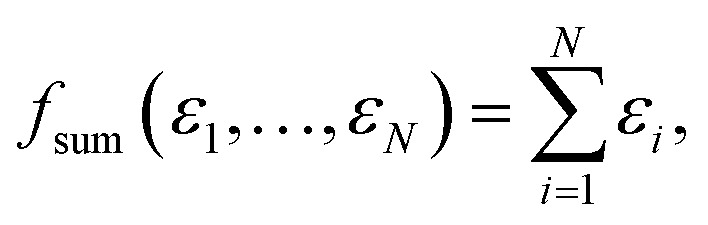
and2
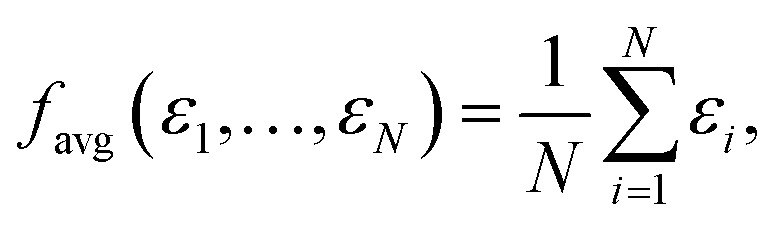
respectively. As discussed above, both of these yield unphysical results for localized intensive properties, however.

The simplest pooling function that potentially shows the correct behavior for such localized properties is max pooling, expressed as:

3 *f*_max_(*ε*_1_,…,*ε*_*N*_) = max({*ε*_1_,…,*ε*_*N*_})

Note that here we are assuming that the target property is the energy of the highest occupied molecular orbital (HOMO). In other cases the min function would be appropriate, *e.g.* for the IE or the lowest unoccupied molecular orbital (LUMO) energy.

While *f*_max_ may have the desired formal properties, it arguably takes things too far since it ultimately makes the predicted molecular or materials property a function of a single atomic contribution. In real interacting systems, even fairly localized orbitals will typically extend over several atoms, however. More importantly, it would be desirable to have a pooling function that is simultaneously adequate both for localized and delocalized properties. A simple way to achieve this is *via* softmax pooling:4
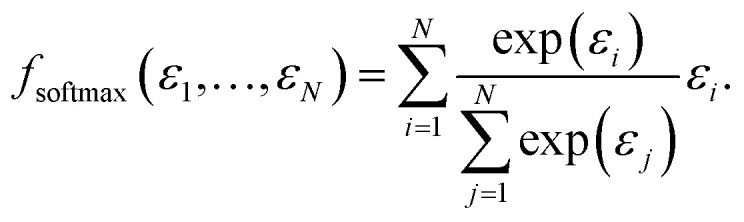


In a fully symmetrical system where each atom has an identical chemical environment this function behaves like average pooling, whereas it behaves more like max pooling in strongly unsymmetric cases like the above mentioned non-interacting water-CO_2_ toy system.

More generally speaking, softmax pooling is just one example of a weighted average, with weights defined as 
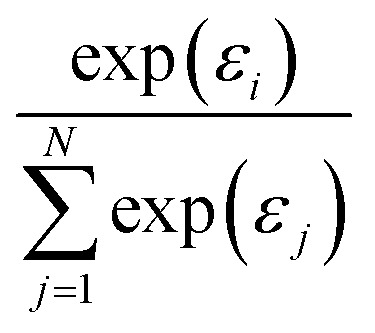
. This assumes that both the target property and its localization can be simultaneously predicted from the scalar outputs *ε*_*i*_. As a more flexible approach, the weights could also be predicted by a second NN, as shown on the bottom of Fig. 1. This leads to the general weighted average (WA) pooling:5
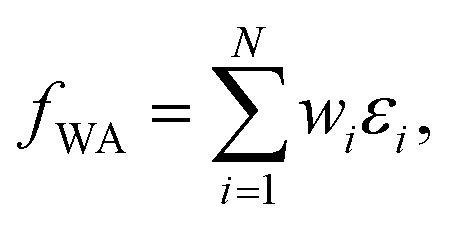
Note that herein the softmax function (see eqn (4)) is used to normalize the outputs of the second NN, so that 
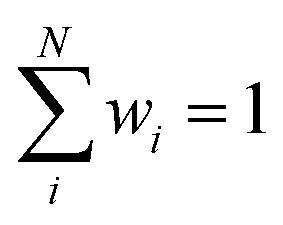
 (see ESI†). This step rigorously enforces size-intensivity of the resulting models.

From a physical perspective it is interesting to consider what the ideal weights in WA pooling should be. For HOMO energy prediction it stands to reason that they should be related to the localization of the orbital. When the HOMO is expressed as a linear combination of atomic orbitals (indexed with *μ*, *ν*), the fraction *l*_*i*_ of the orbital that is localized on a given atom *i* can be obtained as:^38^6
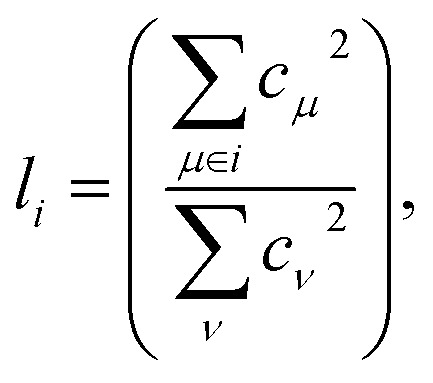
where *c*_*μ*_ are the orbital coefficients in the atomic basis and the upper sum is restricted to all basis functions localized on atom *i*. Based on this, we can define an orbital coefficient based pooling function:7
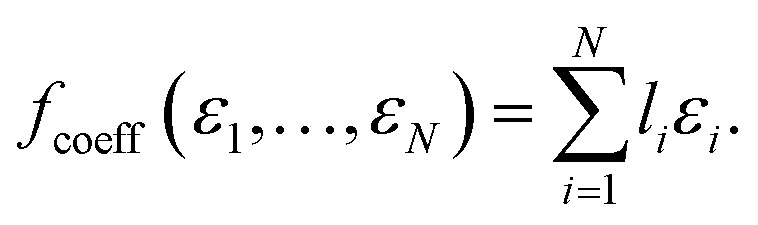


Clearly, this function is of limited practical value for predicting orbital energies though. If the orbital coefficients were known, so would be the corresponding energies. Nonetheless we apply this coefficient pooling function below as a benchmark. In principle, it could also be applied with orbital coefficients from lower level methods, but this is beyond the scope of the current work.

As a practically tractable and computationally efficient approximation to *f*_coeff_, we explore including *l*_*i*_ in the training procedure of WA models. In the resulting Orbital Weighted Average (OWA) approach, the loss function is augmented so that the weights reproduce the orbital localization fractions *l*_*i*_ as closely as possible:8
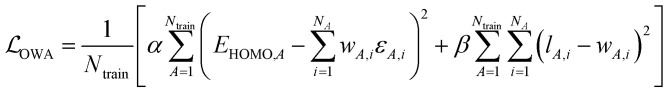
Here, the loss is computed as an average over all *N*_train_ systems *A* in the training set or batch. To clarify this, each of the previously used variables is augmented with an additional index *A* in this equation. The global parameters *α* and *β* determine the relative contributions of orbital energies and localizations to the loss. The latter are optimized for orbital energy prediction on a separate validation set (see ESI†). In contrast, WA models are trained on the same purely orbital energy based loss function as the other models (see ESI†).

It should be noted that sum, average and max pooling have previously been used in the literature, *e.g.* in ref. 24, while the other approaches discussed herein are to the best of our knowledge used for the first time for molecular property prediction. We also note that the simple pooling functions used herein can in principle be replaced by separate neural network components, which try to learn appropriate pooling behaviour from data.^39^ In this case, correct scaling with system size is not rigorously enforced, however.

### 2.3 LocalOrb dataset

**Fig. 2:**
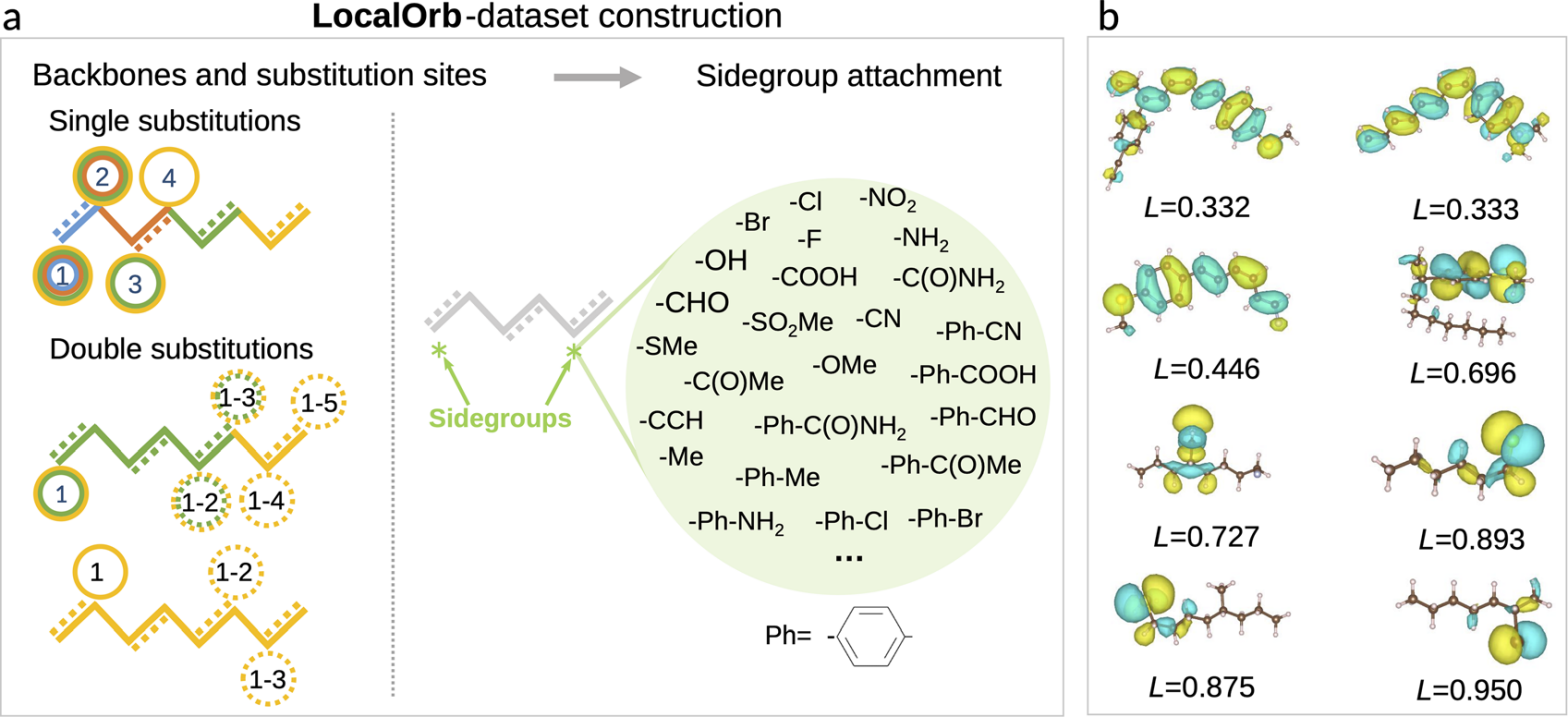
LocalOrb dataset. (a) Illustration of the dataset construction principle, with alkane and conjugated alkene backbones of different length being decorated with one or two sidegroups. Note that only a representative subset of the 41 sidegroups is shown. Substitution sites are separated by at least three carbon atoms to avoid steric clashes. (b) Example molecules from the LocalOrb dataset with HOMO isosurfaces showing the diversity of localized and delocalized orbitals. This is quantified by the orbital localization index *L*, defined in the main text.

Having established a series of pooling functions with desirable formal properties, our next goal is to benchmark how accurately the corresponding models can predict localized electronic properties. As a challenging test case we set out to predict HOMO energies in flexible organic molecules, which span a wide range of localization degrees. Specifically, a set of candidate molecules was generated by substituting 41 functional groups^40^ at predefined positions of alkane or alkene backbones as illustrated in Fig. 2a. The chain length of these backbones varies from two to eight carbon atoms (see ESI† for a definition of all sidegroups and backbones, as well as further details on the dataset). All molecules in this chemical space were enumerated as SMILES strings, using the RDKit package.^41^ Duplicated SMILES were detected and removed from the dataset, resulting in 21 081 unique 2D structures with a maximum of 11 rotatable bonds.

Initial 3D structures were generated from the SMILES strings using the ETKDG method^42^ as implemented in RDKit. Based on these geometries, the CREST^43^ package was used to explore the conformational space of each molecule at the semi-empirical GFN2-xTB level.^44^ Default values were used for all CREST hyperparameters. Final geometries were obtained using the efficient *meta*-GGA composite density functional theory (DFT) method r2SCAN-3c^45^ as implemented in ORCA 5.0.2.^46^ To avoid the well known delocalization errors of semi-local density functionals, accurate orbital energies and coefficients were finally obtained with the range-separated hybrid wB97X-D3 (ref. 47) functional and def2-TZVP^48^ basis set.

Note that the choice of saturated and conjugated backbones and the wide range of electron withdrawing and donating functional groups considered herein ensures a high diversity in the localization of the HOMO for these molecules (see Fig. 2b). This is further exacerbated by their high flexibility, which leads to an additional influence of the specific conformer configurations on orbital localization and energetics.^49^

For training and model evaluation, the 21 081 unique molecules were separated into two categories: to generate the training set, 4000 unique molecules were used. After the corresponding CREST runs, the lowest energy conformer and up to five further randomly selected conformers were used for DFT refinement, yielding 18 784 structures overall. To generate an independent test set, 15 462 of the remaining unique molecules were used. Here only the most stable conformer was refined with DFT for each molecule. This choice was made to maximize the chemical diversity in the test set, since we expect orbital locality to be more strongly influenced by the molecular structure than by the conformation.

### 2.4 Orbital localization index

As we are interested in the performance of the proposed pooling functions for both localized and delocalized HOMOs, a metric for orbital localization in a given molecule is needed. To this end, we can use the orbital localization fractions *l*_*i*_ defined in eqn (6). Specifically, we define the orbital localization index *L* as:9
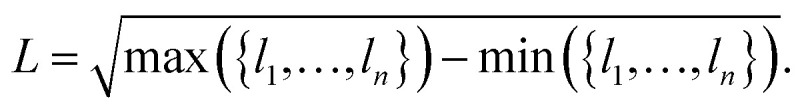
If the HOMO is fully localized on a single atom this yields *L* = 1, whereas *L* = 0 if the HOMO is evenly distributed across all atoms.

While this definition is admittedly somewhat arbitrary, the metric matches our intuitive concept of localization and delocalization rather well, as shown in Fig. 2b. This also illustrates that the LocalOrb dataset indeed covers a highly diverse range of orbital distributions. Based on this we define highly localized orbitals as those with *L* ≥ 0.8 and highly delocalized ones as those with *L* < 0.4.

## 3. Results

### 3.1 Pooling function performance

**Fig. 3:**
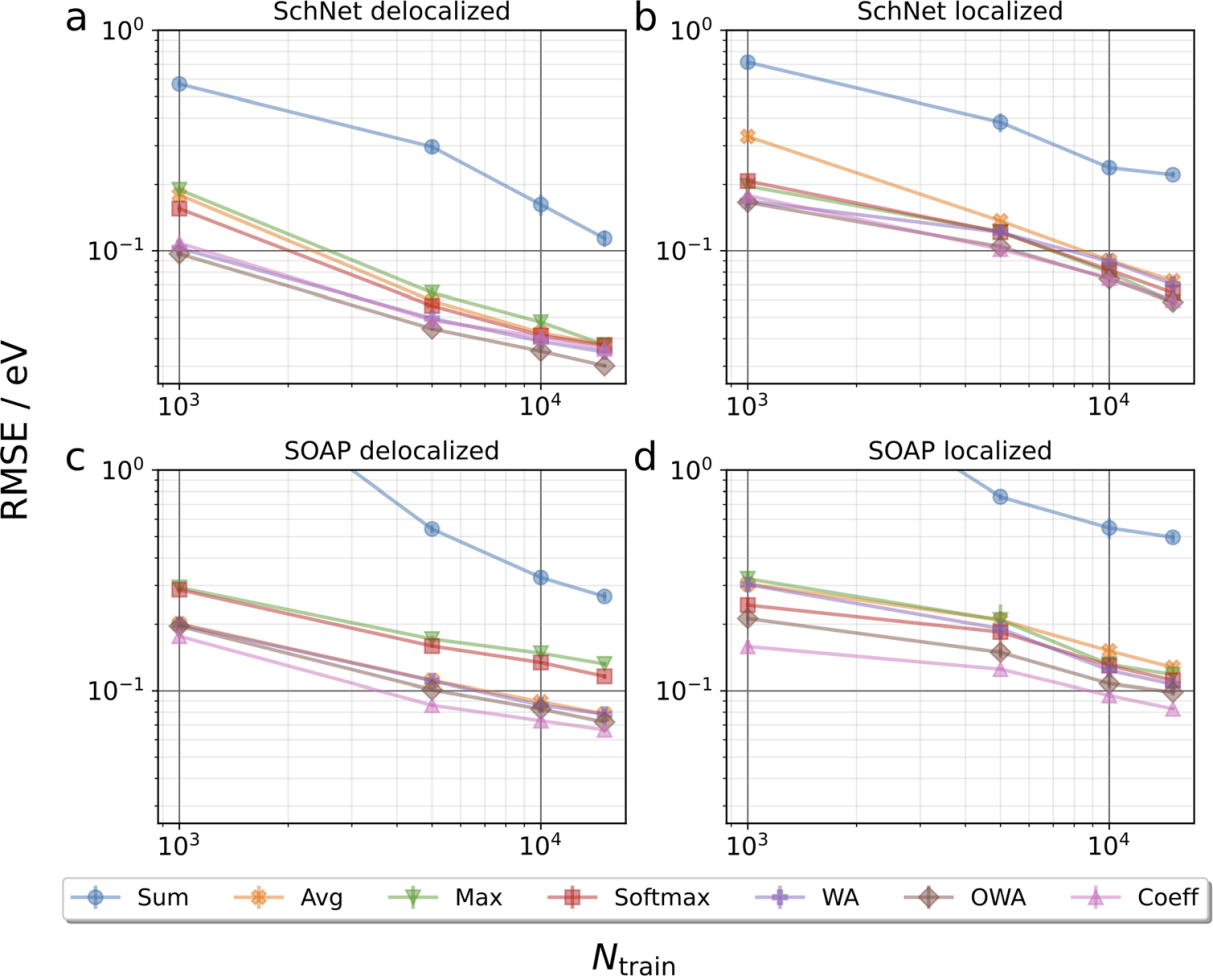
Learning curves for HOMO energy prediction on LocalOrb. The root mean squared errors (RMSEs) of atomistic neural networks based on the SchNet and SOAP representations are shown for test set molecules with particularly delocalized or localized orbitals, as a function of the training set size *N*_train_. Error bars indicate standard deviations over five randomly drawn training sets of the respective size. Note that the WA curve in frame c nearly overlaps with the Avg curve.

Fig. 3 collects learning curves for SchNet and SOAP based models using the pooling functions defined above. Here, subsets of the test set are shown, emphasizing molecules with particularly delocalized (*L* < 0.4, 3867 systems) and localized (*L* ≥ 0.8, 539 systems) orbitals. Learning curves for the full test are shown in Fig. S5.† Directly at first glance this already reveals that localized orbitals are more challenging to predict, though this may be related to the fact that they are less frequent in the training set. Indeed, the performance for localized orbitals is quite sensitive to the number of localized configurations in the training set, as shown in Fig. S6.†

More importantly, the pooling functions are found to have a substantial influence on performance. In all cases, sum pooling displays very large errors. This underscores the importance of using properly intensive pooling functions when predicting orbital energies that has previously been reported.^18,24^ Among the intensive pooling functions the differences are more subtle but still significant. Max pooling performs worst for delocalized systems with softmax being a slight improvement. Meanwhile, the commonly used average pooling tends to perform somewhat better than max and softmax for delocalized systems but worse for localized ones. This is basically in line with our expectations, since average and max are by construction suited for highly delocalized and highly localized orbitals, respectively. Though softmax should in principle represent a compromise between these extremes, it performs quite similarly to max in our tests.

To improve further, we turn to the more sophisticated weighted average approaches. As discussed in the Methods section, coefficient pooling represents a benchmark method in this context, as it incorporates exact information about orbital localization. We find that it indeed yields a significant improvement over average pooling and is among the best methods overall. Perhaps surprisingly, OWA pooling is even better in some cases, although it is formally designed to approximate coefficient pooling. To verify that the improved performance of OWA and WA is not merely due to the larger number of trainable parameters in the pooling function, additional SchNet results for average pooling models with increased embedding dimensions are shown in the ESI.† This reveals that simply increasing the capacity of the networks does not improve the test performance in this case.

As noted above, the OWA model predicts orbital localization with a second neural network, trained on the orbital fractions used in coefficient pooling. Its superior performance is likely due to the fact that both NNs in the model are trained using a joint loss function that depends both on the orbital locations and energies. Consequently, the model can in principle improve the predictive accuracy on energies by deviating from the reference orbital localizations. This additional flexibility is missing in the case of coefficient pooling.

Nevertheless, the orbital fractions provide an important inductive bias for the model. This is illustrated by the fact that WA pooling (which lacks this information) performs somewhat worse than both the OWA and coefficient pooling methods. Overall, OWA is found to be at least as accurate as the coefficient pooling benchmark and much more efficient from a computational perspective. It thus emerges as the pooling function of choice for localized intensive properties.

While not being the main focus of this paper, it is also interesting to compare the performance of the SchNet and SOAP based models. Overall, the SchNet models are found to be somewhat more accurate. This is in contrast to other benchmarks, *e.g.* for atomization energies, where SOAP-based models usually outperform SchNet (particularly for small training sets).^2^ However, it should be emphasized that no hyperparameter optimization of the SOAP representation has been performed herein and that there is no reason to believe that the defaults we used are optimal for orbital energy prediction. A more detailed comparison of SchNet and SOAP is beyond the scope of this paper, however.

It is also notable that the spread among different pooling functions is somewhat larger for SOAP than for SchNet. This is likely due to the fact that the message passing mechanism in SchNet gives some additional flexibility to compensate inadequacies of the pooling functions. In particular, the scalar atomic quantities that are passed to the pooling function are much less local in SchNet than in SOAP. In other words, the message passing scheme performs some preliminary pooling among neighboring atoms. For conciseness we focus on the SchNet models in the following.

### 3.2 Predicting orbital locations

An added benefit of pooling functions like softmax, WA and OWA is that their weights can in principle be interpreted as approximate orbital localization fractions *l*_*i*_. This is particularly pertinent for the OWA approach, where the weights should approximate *l*_*i*_ by design. However, it is also interesting to consider if methods like softmax and WA implicitly learn to predict orbital locations when training on orbital energies alone.

**Fig. 4:**
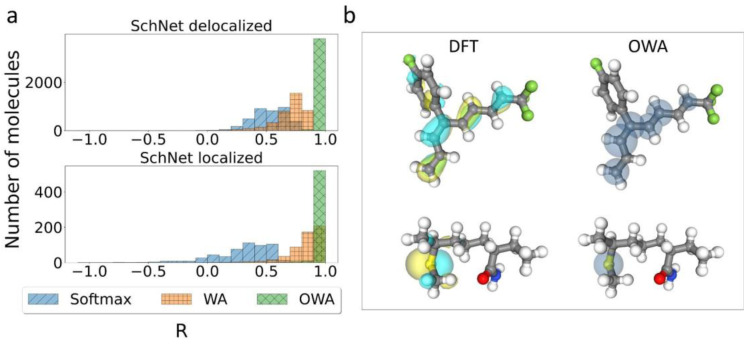
Predicting Orbital Locations. (a) Pearson correlation coefficients *R* between DFT-based orbital localization fractions *l*_*i*_ and machine-learned weights obtained with different pooling functions. The two panels show correlations for particularly delocalized and localized systems, respectively. (b) Visual comparison of DFT orbitals and machine-learned pseudoorbitals obtained with the OWA approach. In the latter, learned weights are visualized as semitransparent spheres.

To quantify this, Pearson correlation coefficients between the learned weights and the DFT-based *l*_*i*_-values were calculated for all molecules in the test subsets used in Fig. 3. The corresponding histograms are shown in Fig. 4a. This confirms that OWA weights indeed represent excellent approximations to the true *l*_*i*_-values, with all correlations being close to 1. The WA method also displays moderate to high correlations, in particular for localized states. In the delocalized case, the spread is somewhat larger but nearly all correlations lie above 0.5. Finally, the softmax method shows the weakest correlations and is particularly bad for the localized cases.

The high correlations between OWA weights and orbital distributions are also shown in Fig. 4b, where the weights are illustrated as semitransparent spheres forming phase-less pseudoorbitals. The OWA NN is thus a bona fide multi-property network that can be used to predict orbital energies and locations on the same footing, with potential applications for organic semiconductors.^50^ The surprisingly good performance of WA in predicting orbital locations (particularly for localized orbitals) also underscores that *l*_*i*_ is the right physical prior for the pooling function in this context. Even if they are not included in the training, the model indirectly (and imperfectly) infers them from the orbital energies.

### 3.3 Application to organic semiconductors

So far we have focused on the intentionally artificial LocalOrb set, which allowed us to study particularly localized and delocalized orbitals in depth. To test whether these insights are transferable to a real chemical application, we now turn to the OE62 dataset.^30^ This set consists of >62 000 organic molecules extracted from crystal structures reported in the Cambridge Crystal Structure Database and was originally composed to screen for potential organic semiconductors.

**Fig. 5:**
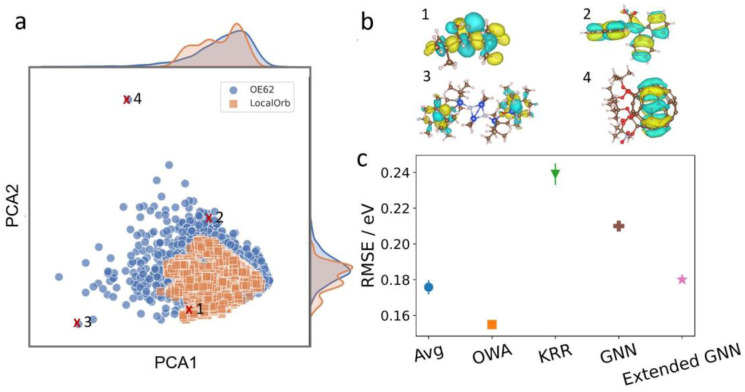
Performance on the OE62 dataset. (a) SOAP-based Kernel principal component analysis plot showing 3000 randomly drawn molecules from the LocalOrb and OE62 datasets. This illustrates the significantly greater structural diversity of OE62. (b) Example molecules from OE62 with HOMO isosurfaces showing different levels of localization. (c) RMSEs of SchNet models using average and OWA pooling compared with previously reported models using Kernel Ridge Regression (KRR),^51^ and Graph Neural Networks (GNNs).^52^ In all cases, 32 000 molecules were used for training, and 10 000 molecules were used as a test set. Where shown, error bars reflect standard deviations over ten randomly drawn training sets.

This dataset is significantly more challenging than LocalOrb, with more structural diversity, a broader size distribution and more chemical elements. This is illustrated *via* a Kernel Principal Component Analysis plot in Fig. 5a.^36^ Here, the LocalOrb set can be seen to cover a subset of the space covered by the OE62 set. Fig. 5b shows four representative molecules from OE62 and the corresponding HOMOs. This confirms that orbital localization is also an important aspect in real organic molecules. Note that since the original OE62 dataset lacks orbital coefficients, these were recomputed for this study (see ESI†).

Because the OE62 dataset has previously been used to train models for HOMO energy prediction, it also allows us to compare the methodology presented herein with the recent literature. To this end, SchNet models with average and OWA pooling were trained on randomly drawn training sets of 32 000 molecules. For robust statistics, this process was repeated ten times for each model and the performance was checked on an unseen test set of 10 000 molecules (see Fig. 5c). This procedure is analogous to the one used in ref. 51, with the best performing model from that paper (using Kernel Ridge Regression and the Many-Body Tensor Representation, MBTR^53^) also shown in Fig. 5c. Both the average and OWA models significantly outperform this baseline (RMSE = 0.24 eV) with RMSEs of 0.18 and 0.15 eV, respectively. Here, the improved performance of OWA is consistent with what we observed for the LocalOrb dataset. We also compare with two more recent graph neural network (GNN) based models from ref. 52, with RMSEs of 0.21 and 0.18 eV, respectively.

This shows that the OWA model displays state-of-the-art performance for HOMO energy prediction on OE62, while also providing orbital localization information, which the other models lack. Importantly, the benefits of the physically motivated OWA pooling function are not restricted to the artificial LocalOrb dataset, but also show up for the realistic and diverse molecules in the OE62 set. As shown in the ESI,† OWA outperforms average pooling across all molecule sizes in OE62, with the biggest improvement for the largest molecules. Overall, OWA can thus be recommended as a robust and physically motivated pooling function for orbital energy prediction.

It should be noted that a series of other orbital energy prediction models have been proposed in the literature, which cannot directly be compared to these results. Most notably, several models were developed to predict machine-learned Hamiltonians, which yield both orbital energies and coefficients upon diagonalization.^20,38,54^ These often focus on a range of occupied and unoccupied orbitals at once, so that they usually do not report HOMO prediction accuracies alone, even when they are tested on OE62.^20^

ML Hamiltonians in many ways are the most physically sound approach to predicting orbital energies and other intensive electronic properties. However, they also represent a significant computational overhead compared to OWA. In particular, their inference costs do not scale linearly with system size, due to the required diagonalization step. To overcome this, ref. 20 uses a constant-size ML Hamiltonian. Here, the correct treatment of isolated supersystems is not guaranteed, however. In our view, pooling functions like OWA therefore fill an important niche, providing physically sound and computationally efficient predictions of localized intensive properties.

## 4. Conclusions

In this contribution, the role of the final aggregation step in predicting localized intensive properties with atomistic neural networks was analyzed. Based on this analysis, a series of physically motivated pooling functions was proposed. To test these functions empirically, we generated the novel LocalOrb dataset, consisting of organic molecules with highly diverse orbital distributions. In this context, the OWA approach, which relies on predicting orbital locations along with their energies was found to be an optimal choice.

The physics-based approach proposed herein has two main advantages over purely data-driven ones. Firstly, it is useful whenever information about the localization of a property is of interest. This is, *e.g.*, the case when modelling organic semiconductors, where orbital locations are relevant for predicting electronic couplings between molecules.^55^ Secondly, rigorously enforcing correct scaling with system size is essential whenever a ML model should be trained on small systems and applied to larger ones, *e.g.* to molecular clusters, crystals or polymers.

More broadly, the current study shows that a physical analysis of the target property based on interesting edge cases like non-interacting subsystems pays real dividends in chemical machine learning. We expect that combining these insights with recent advances in neural network architectures (*e.g.* the NequIP,^56^ GemNet,^57^ or MACE^58^ models) can lead to further improvement in predicting orbital or ionization energies for complex systems.

Finally, the scope of localized intensive properties is in principle much wider than orbital energies and the related quantities discussed herein. For example, defect formation energies, catalytic activities or drug binding affinities display similar characteristics. In future work, we aim to generalize the findings of this study in these directions. In this context, it should be emphasized that localization is a property specific concept. Multi-property networks will thus require multiple weight networks. Furthermore, physical reference values for localization are not always as straightforward to define.

## Data availability

Data and code for this paper are publicly available at https://gitlab.mpcdf.mpg.de/kchen/localized-intensive-property-prediciton.git.

## Author contributions

This project was conceptualized by K. C. and J. T. M. K. C. implemented the concept and conducted the corresponding calculations. Methodological details were worked out by K. C., C. K., B. Q. C., and J. T. M. K. C., K. R. and J. T. M. wrote the manuscript. All authors discussed and revised the manuscript.

## Conflicts of interest

The authors declare no competing financial interests.

## Supplementary Material

SC-014-D3SC00841J-s001

SC-014-D3SC00841J-s002

SC-014-D3SC00841J-s003
