# Supplementary material for: Physics-inspired machine learning of localized intensive properties
Source: Chem Sci. 2023 Apr 10;14(18):4913–22. doi: 10.1039/d3sc00841j (PMC10171074; doi:10.1039/d3sc00841j)
Supplement: SC-014-D3SC00841J-s001 [file SC-014-D3SC00841J-s001.pdf]

Electronic Supplementary Material (ESI) for Chemical Science.
This journal is © The Royal Society of Chemistry 2023

Supporting Information for Physics-Inspired Machine Learning of Localized Intensive Properties

Ke Chen,[1,2,3] Christian Kunkel,[1] Bingqing Cheng,[3] Karsten Reuter,[1,2] and Johannes T. Margraf[1,a)]
[1)] *Fritz-Haber-Institut der Max-Planck-Gesellschaft, Faradayweg 4-6, D-14195 Berlin, Germany*
[2)] *Chair for Theoretical Chemistry and Catalysis Research Center, Technische Universität München, Lichtenbergstraße 4, D-85747 Garching, Germany*
[3)] *Institute of Science and Technology, Am Campus 1, 3400 Klosterneuburg, Austria*

Hyperparameters and Training Procedure. All models are implemented with the `SchnetPack` library. For the SchNet representation, a 128 dimensional embedding is processed by 6 interaction modules using a 5 Ångstrom cutoff. 64 evenly spaced Gaussian functions are used as the radial basis. For the SOAP representation, universal hyperparameters as defined in the ASAP package are used. Fully connected multilayer perceptrons (MLPs) with four hidden layers of 128, 64, 32 and 16 neurons, respectively, are used to generate the atomic outputs ε_i defined in the main manuscript from the SchNet and SOAP representations. Shifted Softplus activation functions are used throughout. For the SOAP models separate networks are trained for each chemical elements, using the `TiledMultiLayerNN` module in `SchnetPack`. The same MLP architecture is used for weight prediction in the *WA* and *OWA* models. Here, the atomic outputs are additionally normalized via a softmax layer. For training, the Adam optimizer is used with a learning-rate decay from 10^{-3} to 10^{-6}. Additionally, the learning rate is reduced by factor 0.8 after 10 epochs without improvement of the validation loss. A batch size of 50 is used for the LocalOrb dataset. This was reduced to 32 for OE62, due to the larger number of elements it contains, which leads to larger memory demands for the SOAP representation.

Loss function for training. As a loss function, we compute the mean squared error between the ML and DFT HOMO energies for the Sum/Avg/Max/Soft/WA pooling functions.

$$\mathcal{L}_{\text{sum}} = \frac{1}{N_{\text{train}}} \sum_{A=1}^{N_{\text{train}}} (E_{\text{HOMO},A} - \sum_{i=1}^{N_A} \varepsilon_i)^2, \tag{1}$$

$$\mathcal{L}_{\text{avg}} = \frac{1}{N_{\text{train}}} \sum_{A=1}^{N_{\text{train}}} (E_{\text{HOMO},A} - \frac{1}{N_A} \sum_{i=1}^{N_A} \varepsilon_i)^2, \tag{2}$$

$$\mathcal{L}_{\text{max}} = \frac{1}{N_{\text{train}}} \sum_{A=1}^{N_{\text{train}}} (E_{\text{HOMO},A} - \max\left([\varepsilon_0, \varepsilon_1, ..., \varepsilon_{N_A}]\right), \tag{3}$$

$$\mathcal{L}_{\text{softmax}} = \frac{1}{N_{\text{train}}} \sum_{A=1}^{N_{\text{train}}} (E_{\text{HOMO},A} - \sum_{i=1}^{N_A} \frac{e^{\varepsilon_i}}{\sum_{j=1}^{N_A} e^{\varepsilon_j}} \varepsilon_i)^2, \tag{4}$$

$$\mathcal{L}_{\text{WA}} = \frac{1}{N_{\text{train}}} \sum_{A=1}^{N_{\text{train}}} (E_{\text{HOMO},A} - \sum_{i=1}^{N_A} w_{A,i} \varepsilon_i)^2, \tag{5}$$

where N_{train} is the number of training samples, N_A is the number of atoms in molecule A. For OWA the loss function is a combination of the HOMO energy error and the deviation between predicted atomic weights and DFT-based orbital localization fractions:

$$\mathcal{L}_{\text{OWA}} = \frac{1}{N_{\text{train}}} \Big[\alpha \sum_{A=1}^{N_{\text{train}}} (E_{\text{HOMO},A} - \sum_{i=1}^{N_A} w_{A,i} \varepsilon_i)^2 + \beta \sum_{A=1}^{N_{\text{train}}} \sum_{i=1}^{N_A} (l_{A,i} - w_{A,i})^2 \Big], \tag{6}$$

[a)] Electronic mail: margraf@fhi-berlin.mpg.de

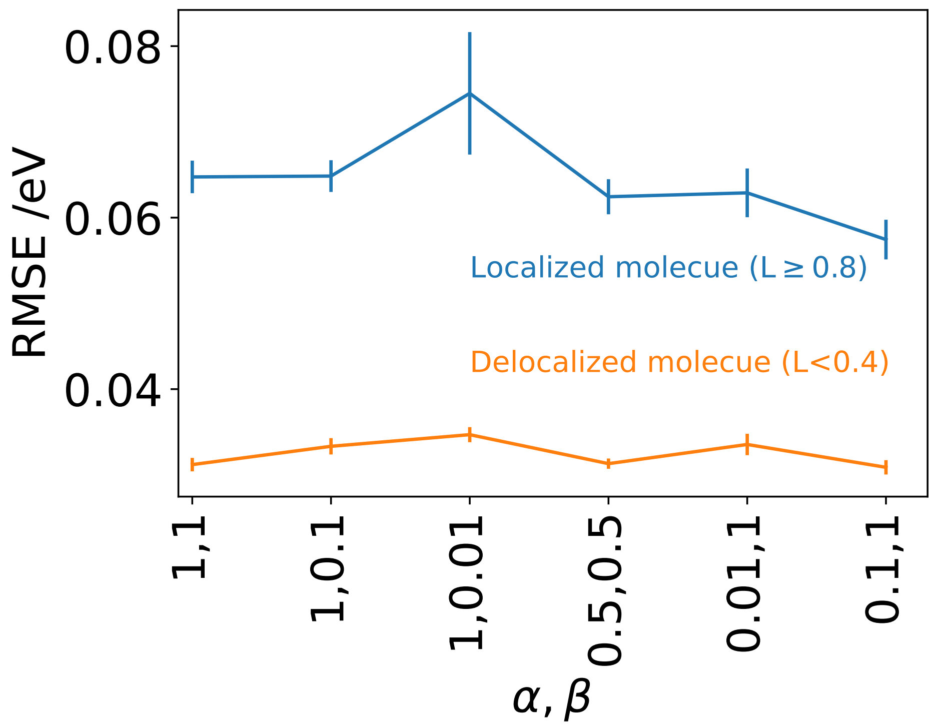

FIG. S1: Grid search for the loss hyperparameters α, β of the OWA method. Finally $\alpha = 0.1$,$\beta = 1$ were selected.

where the coefficients α and β are optimized via grid search (see Fig. S1), $l_{A,i}$ is the orbital localization fraction and $w_{A,i}$ is the predicted atomic weight for atom i in molecule A.

Dataset split. For the LocalOrb learning curves, training subsets (and corresponding validation sets of 2000 molecules) are randomly drawn from the overall training set. We tested the performance for particularly delocalized and localized orbitals in the test set. These are chosen from the overall test set based on the L criteria. Training was repeated five times for each pooling function with a maximum number of 200 epochs for SchNet NNs (and 100 epoch for SOAP NNs). The final results are the average performance of these five models. We note that for the two molecular representations, SchNet and SOAP, identical training, validation and test sets were used.

For the OE62 dataset, we also carried out single point calculations by ORCA 5.0.2 for the equilibrium structures optimized at the hybrid PBE functional. As for LocalOrb, the wB97X-D3 functional and def2-TZVP basis set were employed to perform the DFT single-point calculations to obtain the orbital information. For the general performance comparison as described in the main text, in order to make direct comparison with the results reported by Stucke et al, 32000/5000/10000 training/validation/test set were randomly selected for each model. To analyze the predictive performance for delocalized and localized orbitals, the localized/delocalzied test sets were choosen from the rest of the OE62 dataset according to the $L \geqslant 0.8$ and $L < 0.4$ criteria, leading to 713/5310 systems in the localized/delocalized test sets, respectively.

[1] A. Stuke, M. Todorović, M. Rupp, C. Kunkel, K. Ghosh, L. Himanen, and P. Rinke, "Chemical diversity in molecular orbital energy predictions with kernel ridge regression," J. Chem. Phys. **150**, 204121 (2019).

[2] O. Rahaman and A. Gagliardi, "Deep learning total energies and orbital energies of large organic molecules using hybridization of molecular fingerprints," J. Chem. Inf. Model. **60**, 5971–5983 (2020).

Backbone	Sidegroups
CC(R)	F,Cl,Br
CC(R)CC	CF_3
C(R)CCC	CN
CCC(R)CCC	NO_2
C(R)CCCCC	CHO
C(R)CCCC(R)C	COOH
C(R)CCCCC(R)	$COCH_3$
C(R)CCCCCCC	$CONH_2$
CC(R)CCCCCC	C=-CH
CCC(R)CCCCC	$SOOCH_3$
CCCC(R)CCCC	CH=NH
CC(R)CCCC(R)CC	OH
C(R)CCCCC(R)CC	OCH_3
C(R)CCCC(R)CCC	NH_2
C(R)CCCCCC(R)C	$NCCH_3$
C(R)CCCCCCC(R)	CH_3
C=C(R)	$NCOCH_3$
C=C(R)C=C	SCH_3
C(R)=CC=C	c1ccc(F)cc1
C=CC(R)=CC=C	c1ccc(Cl)cc1
C(R)=CC=CC=C	c1ccc(Br)cc1
C(R)=CC=CC(R)=C	c1ccc([C](F)(F)F)cc1)
C(R)=CC=CC=C(R)	c1ccc([C]=N)cc1
C(R)=CC=CC=CC=C	c1ccc(N(=O)(=O))cc1
C=C(R)C=CC=CC=C	c1ccc([CH]=O)cc1
C=CC(R)=CC=CC=C	c1ccc([C](=O)O)cc1
C=CC=C(R)C=CC=C	c1ccc(C(=O)C)cc1
C=C(R)C=CC=C(R)C=C	c1ccc(C(=O)N)cc1
C(R)=CC=CC=C(R)C=C	c1ccc(C=C)cc1
C(R)=CC=CC(R)=CC=C	c1ccc(S(=O)(=O)C)cc1
C(R)=CC=CC=CC(R)=C	c1ccc(C=N)cc1
C(R)=CC=CC=CC=C(R)	c1ccc([OH])cc1
	c1ccc(OCH_3)cc1
	c1ccc(NH_2)cc1
	c1ccc($NCCH_3$)cc1
	c1ccc(CH_3)cc1
	c1ccc($NCOCH_3$)cc1
	c1ccc(SCH_3)cc1
	c1ccccc1

TABLE I: Backbones and sidegroups for LocalOrb dataset.

Method	RMSE (eV)
Sum (SchNet)	0.411 ± 0.089
Average(SchNet)	0.176 ± 0.004
Max(SchNet)	0.174 ± 0.003
Softmax(SchNet)	0.168 ± 0.003
Coeff(SchNet)	0.161 ± 0.001
WA	0.164 ± 0.002
OWA	0.155 ± 0.002
Ref KRR-MBTR[1]	0.239 ± 0.006
Ref GNN[2]	0.209
Ref GNN-MBTR-MD[2]	0.180

TABLE II: The RMSE of OE62 with different methods testing on 10k dataset under 32k training data.

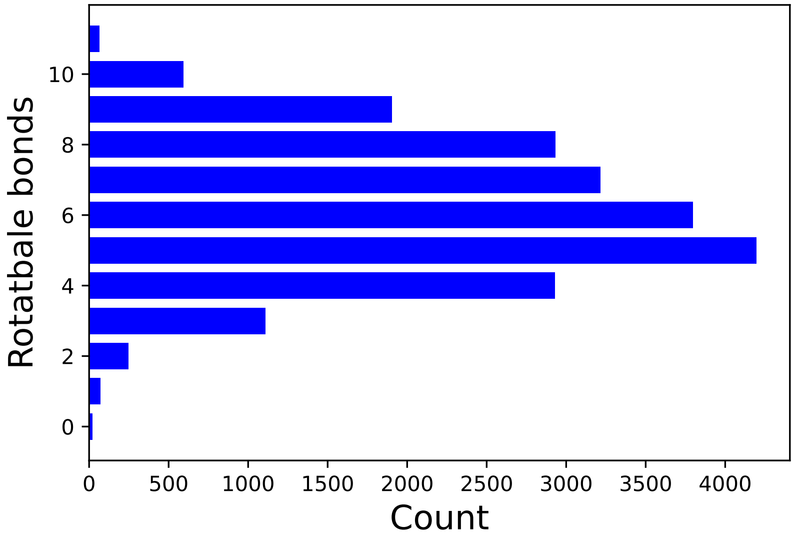

FIG. S2: Rotatable bonds distribution for our own dataset LocalOrb.

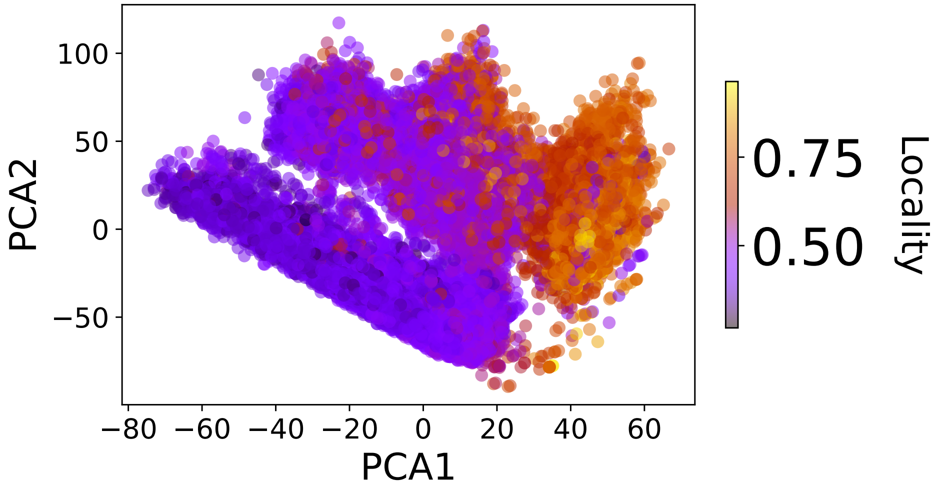

FIG. S3: A kPCA visualization of the LocalOrb dataset colored according to the locality criterion L.

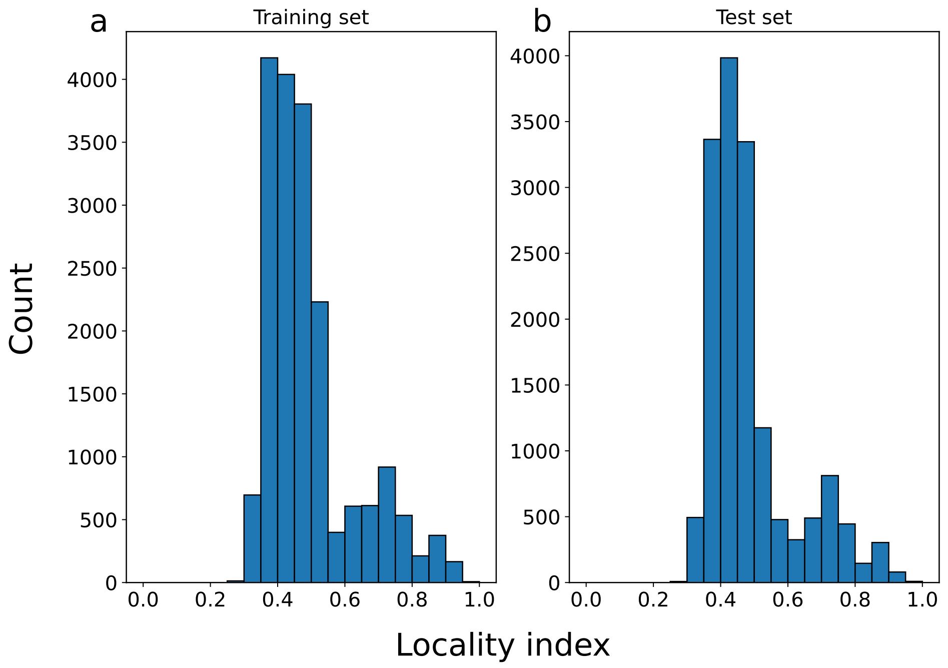

FIG. S4: The Locality index distribution in the LocalOrb training and test set.

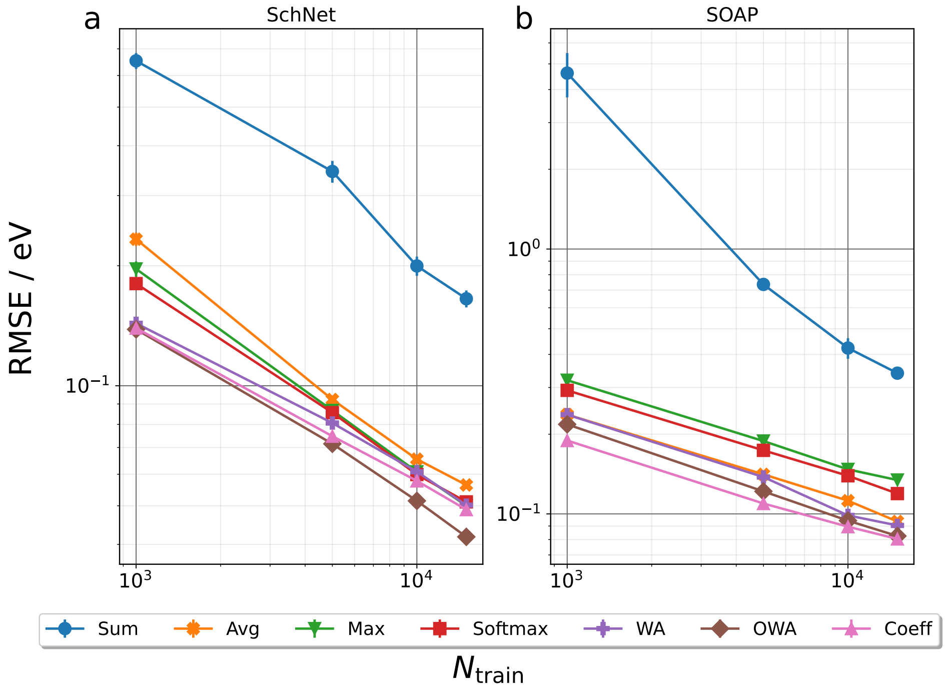

FIG. S5: The learning curve for the full LocalOrb test set.

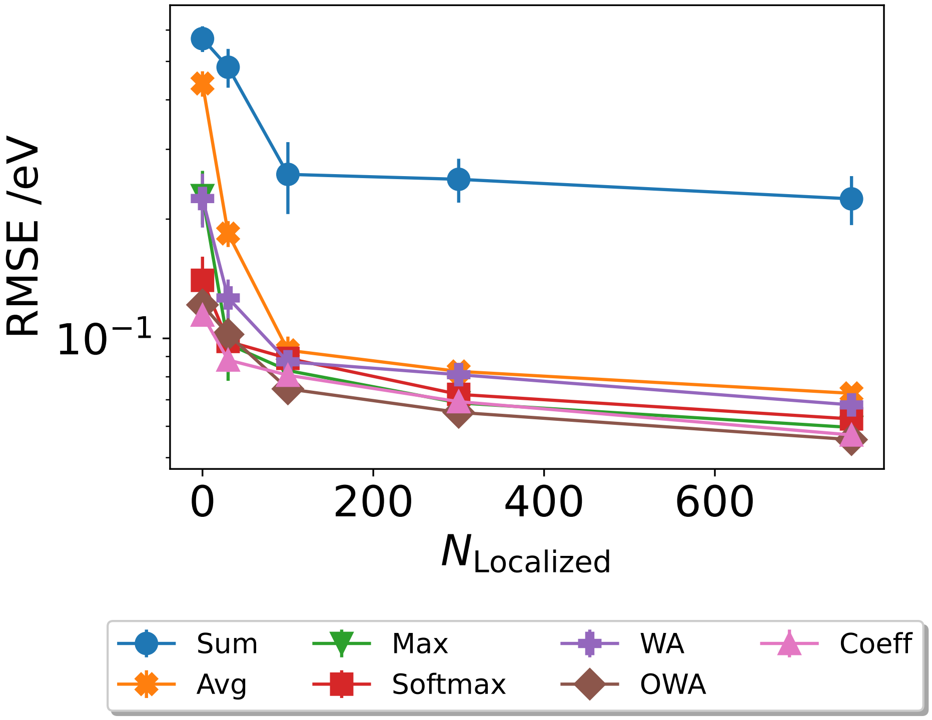

FIG. S6: Effect of the number of highly localized orbitals in training set on predictive accuracy for localized orbitals. The training set size kept constant at 15,000 configurations for this experiment.

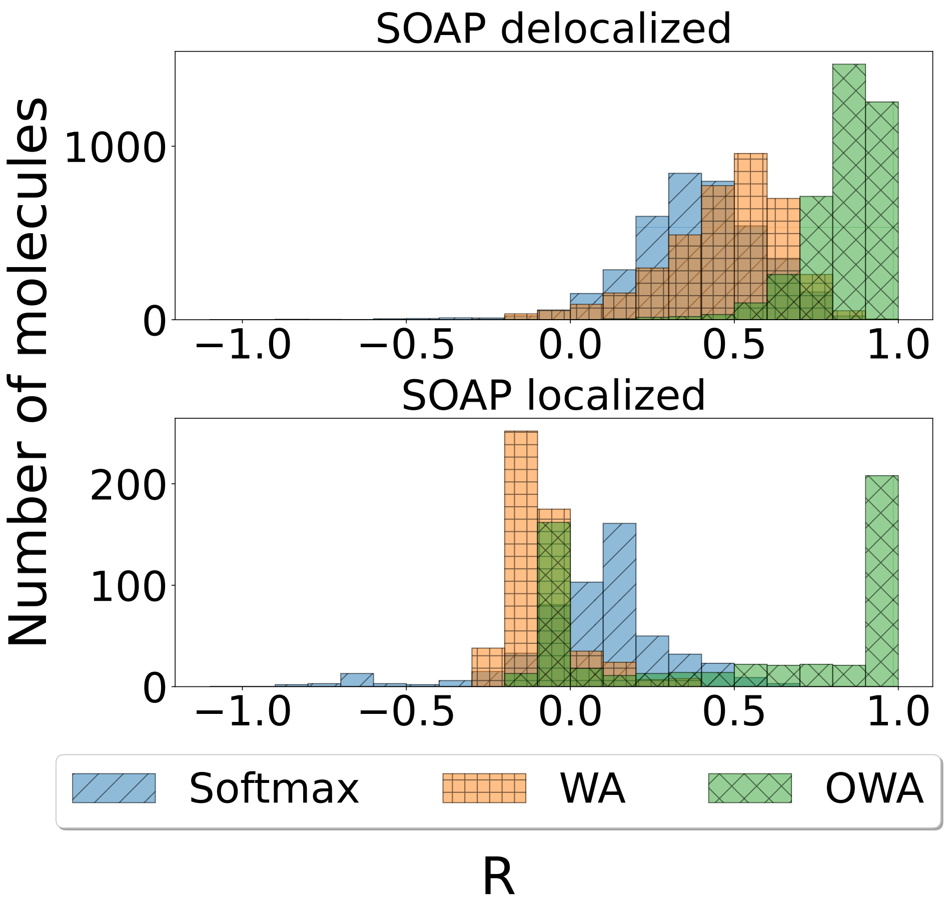

FIG. S7: Pearson correlation coefficients R between DFT-based orbital localization fractions l_i and machine-learned weights obtained with different pooling functions for SOAP representation.

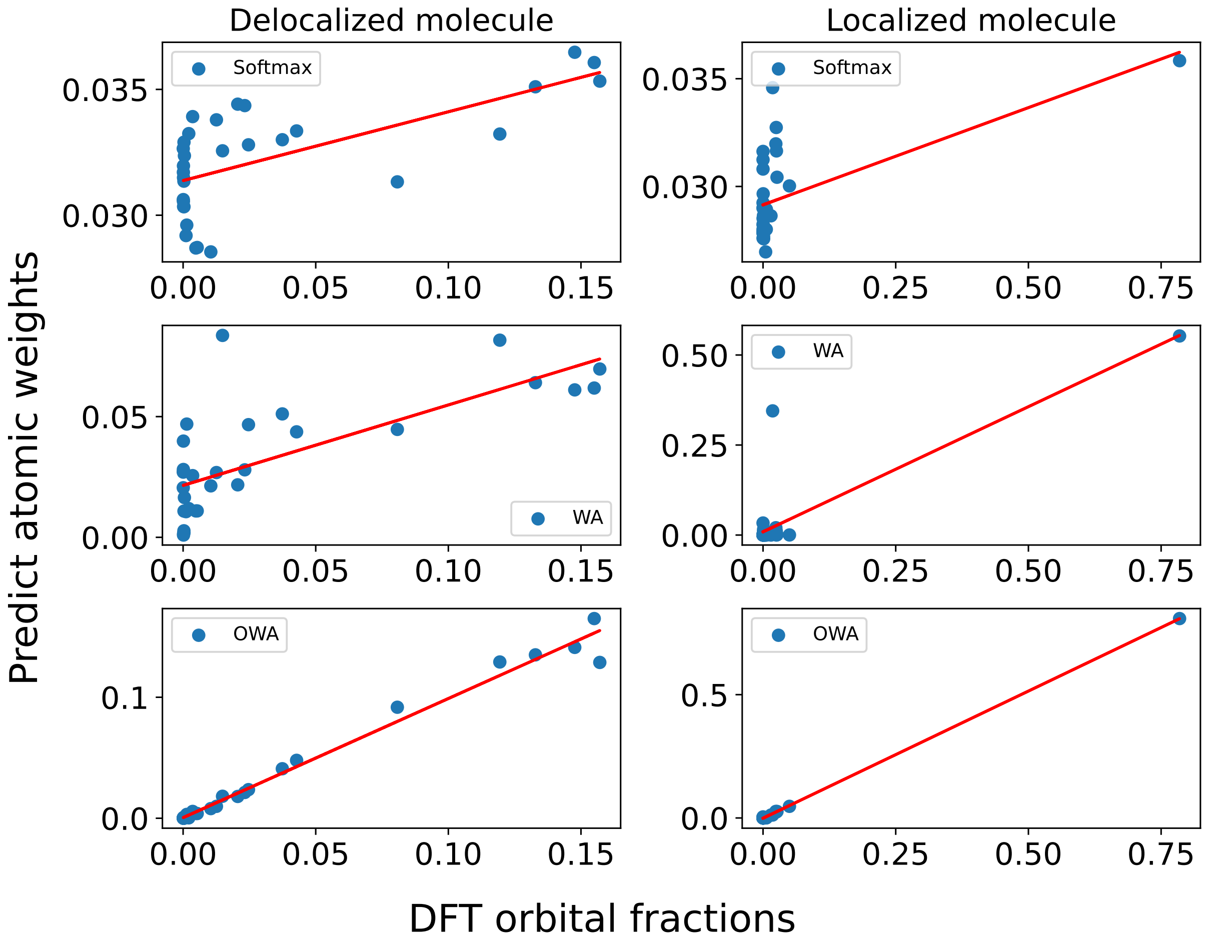

FIG. S8: Exemplary correlations between DFT-based orbital localization fractions l_i and machine-learned weights w_i obtained with different pooling functions for a delocalized and a localized molecule using the SchNet representation. These molecules are depicted in Figure 4b.

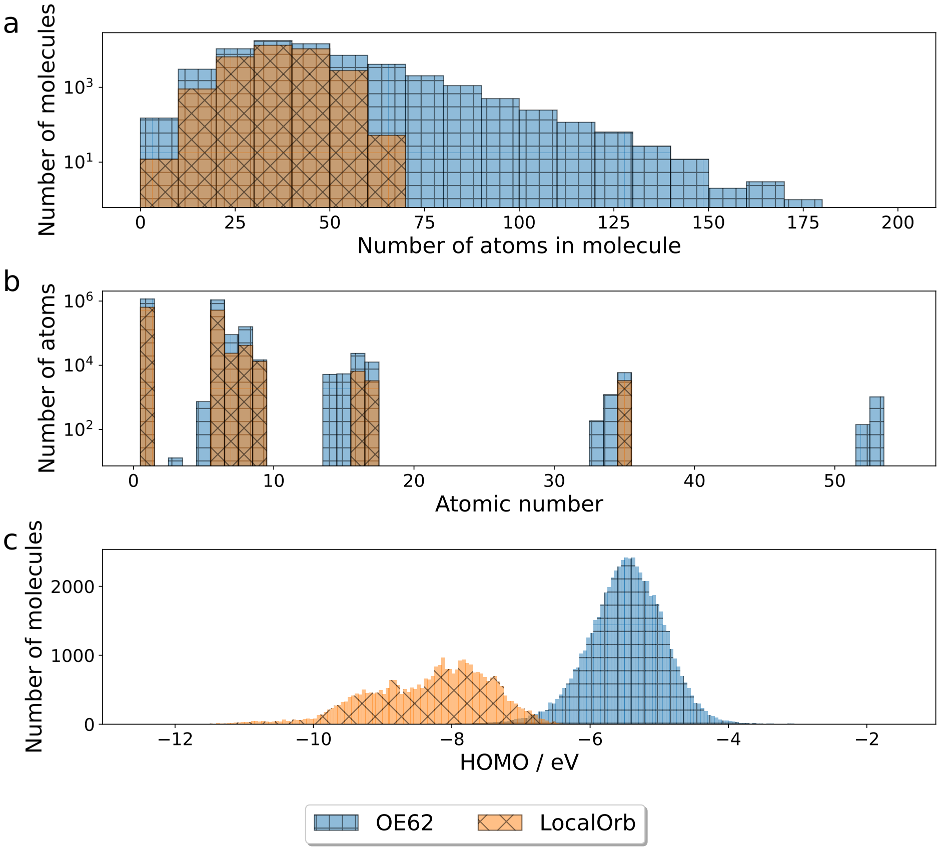

FIG. S9: Comparison of the OE62 and LocalOrb datasets with respect to a) the number of atoms per molecule, b) the elemental distribution, and c) the HOMO energy distribution.

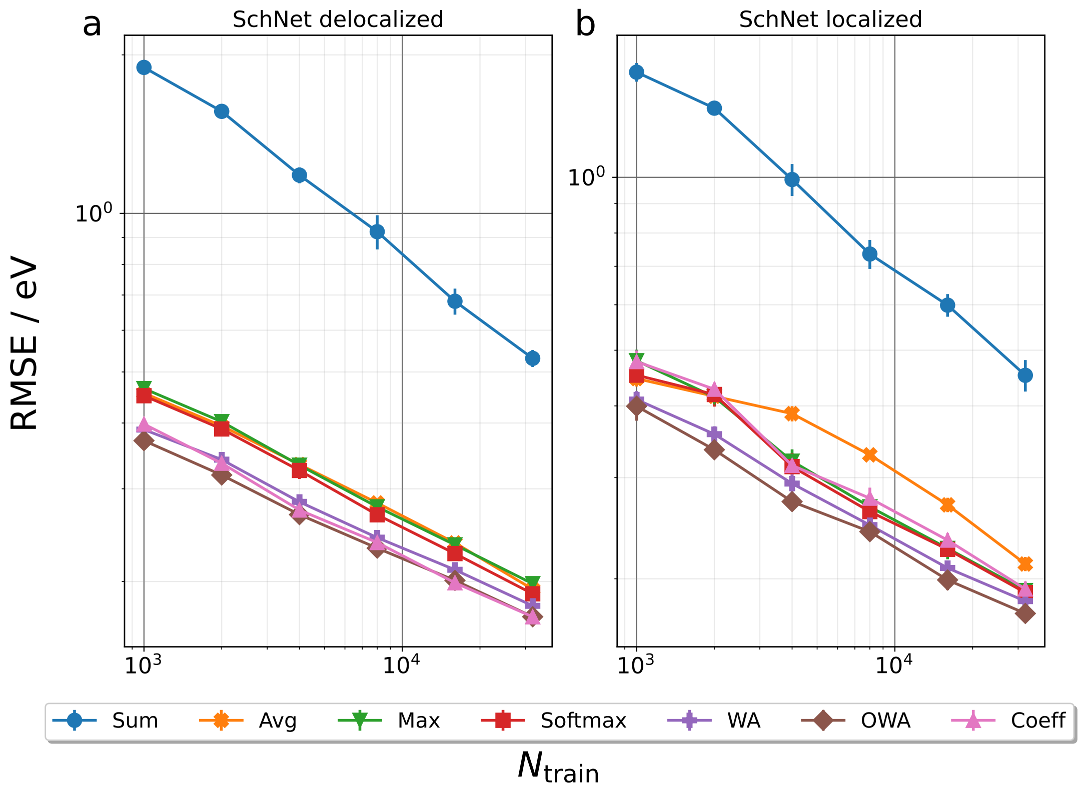

FIG. S10: Learning curve of OE62 for a) delocalized and b) localized molecules.

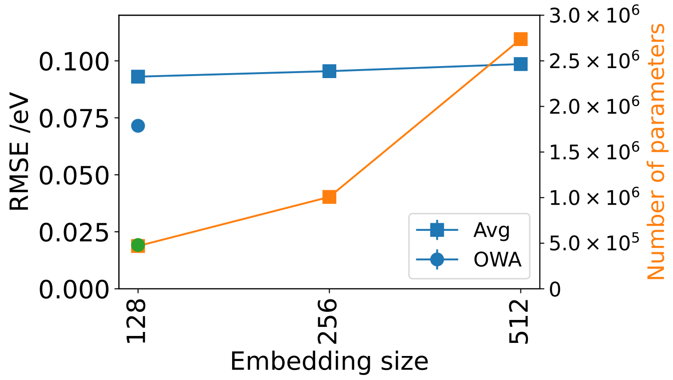

FIG. S11: LocalOrb test set performance for an average pooling model with increasing size of the SchNet embedding vector (blue). The number of trainable parameters in the NN models is shown in orange. Training/validation set sizes are 5000/2000. For reference, the *OWA* model with the embedding size (128) used in the main manuscript is also shown, with the corresponding number of parameters in green.

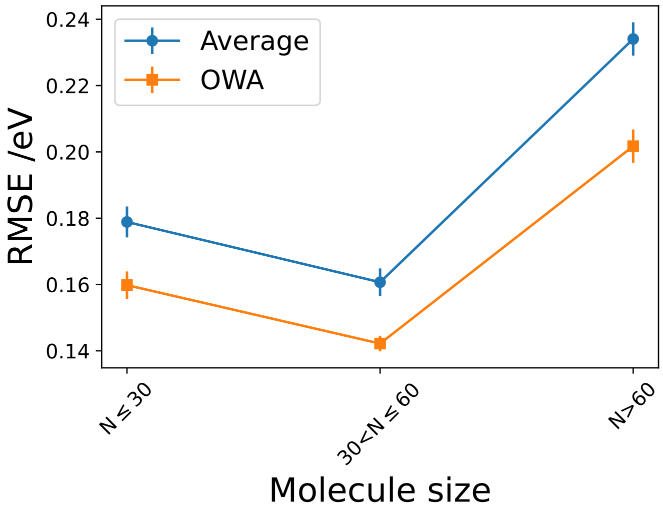

FIG. S12: OE62 test set errors binned by molecule size (in terms of the number of atoms N) for average and OWA pooling. Training/validation set sizes are 32,000/5,000. Note that the training and test sets are not uniform with respect to molecule size. The bins contribute 26%, 62% and 12% of the molecules to the test set, respectively.
